# Circular Stricture—Enormous Distension of the Bladder

**Published:** 1842-07-16

**Authors:** 


*Circular Stricture—Enormous Distension of the Bladder.*—M. Berard presented to the Anatomical Society a bladder enormously distended, and reaching up to the umbilicus; there was a ring stricture at about three inches from the external orifice of the urethra. The bottom of the bladder presented a number of small granulations, like millet seeds; these are nothing but mucous follicles, which are invisible in a healthy condition of the organ.

